# Disease-Associated Signatures Persist in Extracellular Vesicles from Reprogrammed Cells of Osteoarthritis Patients

**DOI:** 10.3390/ijms26030870

**Published:** 2025-01-21

**Authors:** María Piñeiro-Ramil, Iván Gómez-Seoane, Ana Isabel Rodríguez-Cendal, Clara Sanjurjo-Rodríguez, Selva Riva-Mendoza, Isaac Fuentes-Boquete, Javier De Toro-Santos, José Señarís-Rodríguez, Silvia Díaz-Prado

**Affiliations:** 1 Grupo de Investigación en Terapia Celular y Medicina Regenerativa, Instituto de Investigación Biomédica de A Coruña (INIBIC), Fundación Pública Gallega de Investigación Biomédica INIBIC, Complexo Hospitalario Universitario de A Coruña (CHUAC), Servizo Galego de Saúde (SERGAS), 15006 A Coruña, Spain; maria.pramil@udc.es (M.P.-R.); ivan.gomez.seoane@sergas.es (I.G.-S.); ana.rodriguezc@udc.es (A.I.R.-C.); i.fuentes@udc.es (I.F.-B.); javier.toro@udc.es (J.D.T.-S.); jose.senaris.rodriguez@sergas.es (J.S.-R.); 2 Grupo de Investigación en Terapia Celular y Medicina Regenerativa, Departamento de Fisioterapia, Medicina y Ciencias Biomédicas, Facultad de Ciencias de la Salud, Universidade da Coruña (UDC), 15006 A Coruña, Spain; 3 Centro Interdisciplinar de Química y Biología (CICA), Universidade da Coruña (UDC), 15008 A Coruña, Spain; 4 Grupo de Investigación en Reumatología (GIR), Instituto de Investigación Biomédica de A Coruña (INIBIC), Fundación Pública Gallega de Investigación Biomédica INIBIC, Complexo Hospitalario Universitario de A Coruña (CHUAC), Servizo Galego de Saúde (SERGAS), 15006 A Coruña, Spain; selva.riva.mendoza@sergas.es; 5 Centro de Investigación Biomédica en Red de Bioingeniería, Biomateriales y Nanomedicina (CIBER-BBN), 28029 Madrid, Spain; 6 Servicio de Reumatología, Complexo Hospitalario Universitario de A Coruña (CHUAC), Servizo Galego de Saúde (SERGAS), 15006 A Coruña, Spain; 7 Servicio de Cirugía Ortopédica y Traumatología, Complexo Hospitalario Universitario de A Coruña (CHUAC), Servizo Galego de Saúde (SERGAS), 15006 A Coruña, Spain

**Keywords:** induced pluripotent stem cells (iPSCs), mesenchymal stromal cells (MSCs), mesenchymal-like cells (MLCs), small extracellular vesicles (sEVs), proteomic analysis, cartilage degradation, regenerative medicine, autologous cell therapy

## Abstract

Osteoarthritis (OA) is a prevalent joint disorder that lacks effective therapies to halt cartilage degeneration. Mesenchymal stromal cell (MSC)-derived small extracellular vesicles (sEVs) are being investigated as promising chondroprotective agents. Compared to primary MSCs, induced pluripotent stem cell (iPSC)-derived MSCs (MLCs) offer superior scalability and enhanced paracrine activity. The aim of this study was to explore the feasibility of using autologous MLC-derived sEVs as a potential therapeutic strategy for OA through the analysis of their protein cargo. iPSCs from an OA patient and a healthy donor were differentiated into MLCs. sEVs were isolated from these MLCs and characterized, with a particular focus on their protein cargo. Both iPSC lines were successfully differentiated into MLCs, which secreted sEVs with comparable size distributions and yields. The analysis of differentially expressed proteins revealed a high abundance of proteins associated with OA pathology and cartilage degradation in sEVs from OA MLCs compared to those from healthy MLCs. The persistence of OA-associated protein signatures in autologous MLC-derived sEVs may limit their therapeutic efficacy. These findings underscore the importance of carefully evaluating disease-specific protein profiles in sEVs for regenerative applications.

## 1. Introduction

Osteoarthritis (OA) is a chronic rheumatic disease characterized by the progressive breakdown of articular cartilage, leading to pain, stiffness, and a substantial reduction in quality of life. As the most prevalent age-related joint disorder, the incidence of OA is rising rapidly with the aging population. This condition is frequently triggered by joint injuries which activate maladaptive repair mechanisms and pro-inflammatory pathways, disrupting cartilage homeostasis and accelerating cartilage degeneration [1]. The prevalence of OA is influenced by factors such as physical activities, musculoskeletal injuries, obesity, and gender. Intense physical activity and prior joint injuries significantly elevate OA risk, while obesity exacerbates joint degradation through mechanical and inflammatory mechanisms [2,3]. Additionally, hormonal factors and smaller joint dimensions contribute to the higher prevalence of OA in women [4]. Despite extensive research efforts to identify effective therapeutic strategies for OA, no current treatment reliably regenerates articular cartilage in clinical settings [5].

In this context, mesenchymal stromal cells (MSCs) have been extensively investigated due to their immunomodulatory capacity and multi-differentiation potential. Initially believed to home injured tissues, engraft, and differentiate, recent research has revealed that MSCs exert their therapeutic effects on OA joints mainly through paracrine signaling. Notably, MSC-derived small extracellular vesicles (sEVs) have demonstrated regenerative and immunomodulatory properties comparable to their parent cells [6,7,8]. Given their lower immunogenicity and reduced safety concerns, these particles represent a promising alternative to traditional cell-based therapies for cartilage regeneration [9].

Interestingly, Sanjurjo et al. (2021) demonstrated that sEVs derived from MSCs obtained from OA patients could enhance chondrocyte viability and proliferation to a degree comparable to sEVs derived from MSCs from healthy donors [10], suggesting that autologous MSCs may serve as a viable source of sEVs with therapeutic potential in OA [11]. However, the clinical application of MSC-derived sEVs is constrained by the heterogeneity, limited availability, and restricted proliferative capacity of these cells [12,13]. In contrast, induced pluripotent stem cell (iPSC)-derived MSCs, commonly termed mesenchymal-like cells (MLCs), offer a promising alternative for obtaining autologous MSCs in clinically relevant quantities with minimal invasiveness [11,14]. Furthermore, MLCs have demonstrated enhanced paracrine activity and stronger immunomodulatory effects compared to donor-matched primary MSCs [15].

Additionally, pre-clinical studies have demonstrated that MLC-derived sEVs exhibit immunomodulatory and chondroprotective effects comparable to those of primary MSC-derived sEVs [16,17,18,19]. Nevertheless, little is known about their cargo and underlying mechanisms of action. In this study, we generated MLCs from two iPSC lines—one derived from an OA patient [20] and one from a healthy donor [21]—and sEVs from both “OA” and “healthy” MLCs were obtained and characterized. To assess the potential of MLCs as an autologous source of sEVs for OA therapy, we performed a comparative proteomic analysis of sEVs from the two MLC types. The analysis of differentially expressed proteins revealed a high abundance of OA-associated proteins among those upregulated in sEVs from OA MLCs. This finding suggests that, despite cellular reprogramming and re-differentiation, iPSC-derived MLCs and their sEVs retain the OA-specific protein signatures of the donor, which might have significant implications for autologous sEV-based regenerative therapies.

## 2. Results

### 2.1. iPSC-Derived Cells Exhibit a Mesenchymal-like Phenotype

**Figure 1:**
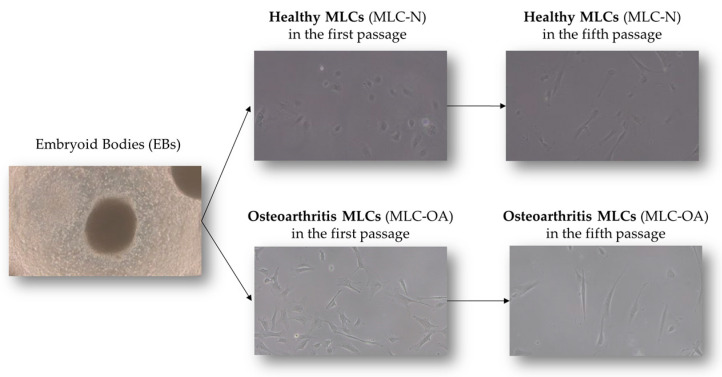
Micrographs showing the phases of differentiation of healthy (N) and pathological (OA) iPSC lines into mesenchymal-like cells (MLCs). Magnification: 4× (EBs), 10× (MLCs).

Both iPSC lines were successfully differentiated into MLCs through embryoid body formation. In general, MLCs exhibited a more polygonal shape during the first passage, gradually adopting a fibroblast-like morphology characteristic of MSCs by the fifth passage (Figure 1).

The expression levels of five mesenchymal surface markers (CD29, CD44, CD73, CD90, and CD105) and two hematopoietic markers (CD34 and CD45) were determined in both MLC types, healthy (MLC-N) and pathological (MLC-OA), across three different passages. More than 80% of the cells were positive for the mesenchymal markers CD29, CD44, CD73, and CD90, while CD105 expression was less than 50%. Positivity for the hematopoietic markers CD34 and CD45 was below 5% (Table 1). No significant expression differences were found between MLC-N and MLC-OA for any of the surface markers analyzed (Supplementary Table S1).

### 2.2. MLCs Exhibit Osteogenic and Chondrogenic Potential but Lack Adipogenic Capacity

**Figure 2:**
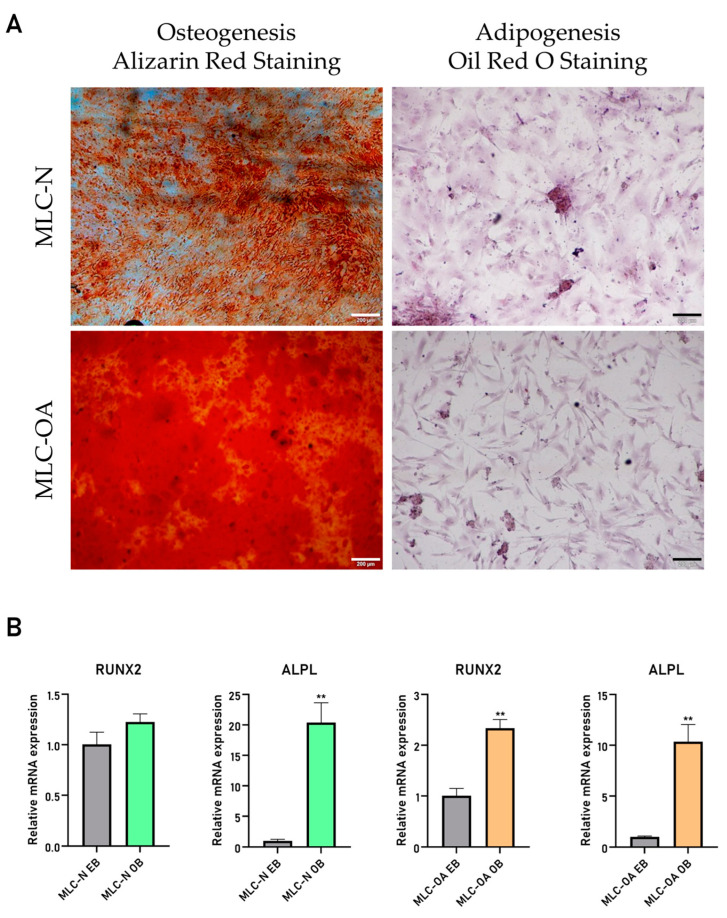
(**A**) Alizarin Red and Oil Red O staining of MLCs after 21 days of osteogenic and adipogenic induction, respectively. Scale bar: 200 µm. (**B**) Relative gene expression of Runt-related transcription factor 2 (RUNX2) and alkaline phosphatase (ALPL) in MLC-N and MLC-OA after 21 days of culture in basal medium (EB) and osteogenic medium (OB). **, *p* ≤ 0.01.

Both types of MLCs differentiated into osteoblasts after 21 days of induction, as demonstrated by Alizarin Red staining, with MLC-OA exhibiting stronger mineralization than MLC-N (Figure 2A). Moreover, the gene expression analysis showed an upregulation of the bone-forming transcription factor Runt-related transcription factor 2 (RUNX2) and the mineralization-associated protein alkaline phosphatase (ALPL) (Figure 2B). On the contrary, no lipid droplets were detected by Oil Red O staining in either MLC type after 21 days of adipogenic induction (Figure 2A).

**Figure 3:**
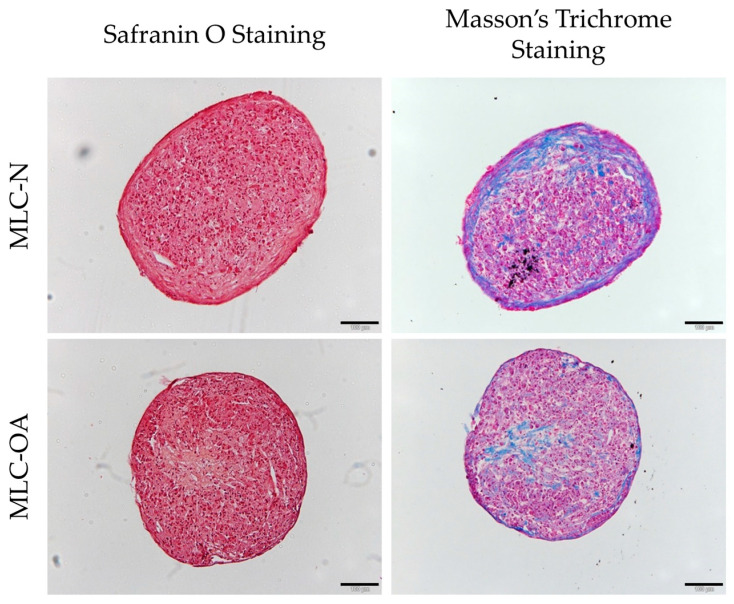
Safranin O and Masson’s Trichrome staining of MLC micromasses after 21 days of culture in chondrogenic medium. Scale bar: 100 µm.

Both MLC types formed dense, round aggregates when subjected to a micromass culture in the presence of chondrogenic *stimuli*. The resulting micromasses exhibited high cell density and the deposition of an extracellular matrix containing collagen (shown in blue in Masson’s Trichrome staining) and proteoglycans (shown in reddish-orange in Safranin O staining). The formation of an outer fibrous layer surrounding the cells in the core of the micromass is particularly evident in MLC-N (Figure 3).

### 2.3. Healthy and OA MLCs Secrete sEVs with Similar Characteristics

**Figure 4:**
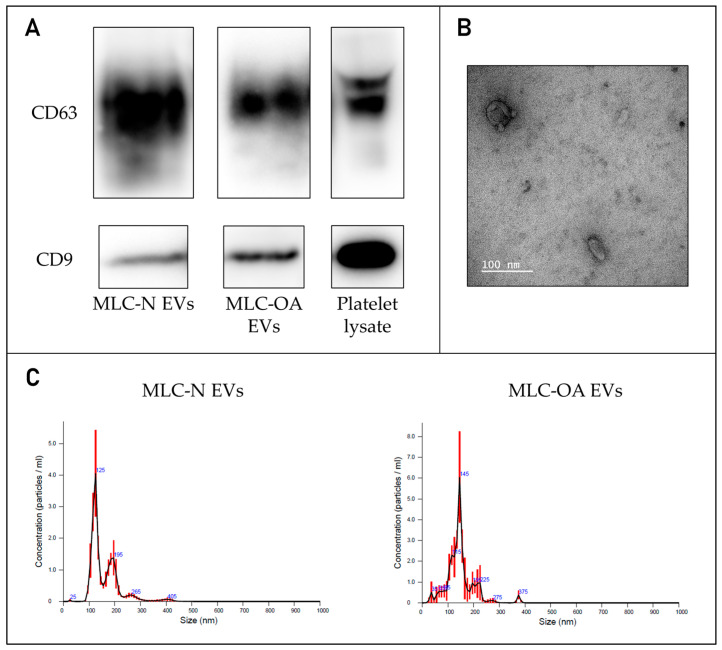
(**A**) Western blotting of the extracellular vesicle markers CD9 and CD63 in sEVs derived from MLC-N and MLC-OA, with platelet lysate as a positive control. (**B**) Morphology of MLC-OA-derived sEVs observed by transmission electron microscopy (TEM). Scale bar: 100 nm. (**C**) Size distribution of MLC-derived sEVs, as measured by nanoparticle tracking analysis (NTA).

MLC-derived sEVs expressed the vesicle surface markers CD9 and CD63 (Figure 4A) and exhibited a characteristic cup-shaped morphology (Figure 4B). Both populations of particles fell within the typical size range of sEVs, with sEVs derived from MLC-OA displaying a slightly larger size (mode: 146.8 ± 3.8 nm) compared to those derived from MLC-N (mode: 119.4 ± 3.1 nm) (Figure 4C). Particle yields were comparable, ranging from 1.27 × 10^9^ to 1.90 × 10^9^ particles per mL of conditioned media for MLC-N and MLC-OA, respectively.

### 2.4. Upregulated Proteins in OA MLC-Derived sEVs Are Associated with OA Pathology

**Figure 5:**
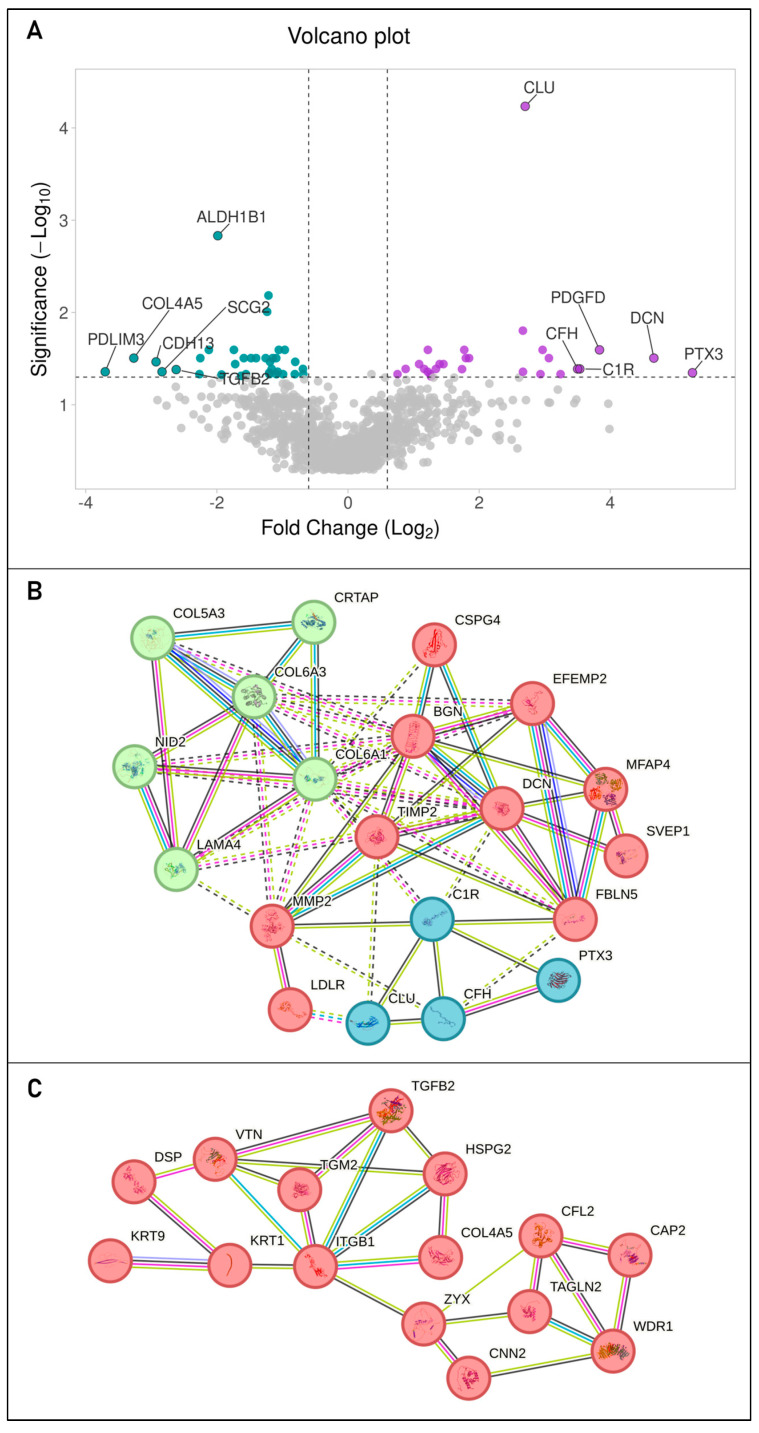
(**A**) Volcano plot showing the proteins downregulated (teal dots) and upregulated (purple dots) in MLC-OA sEVs compared to MLC-N sEVs, with a threshold of ±0.6 log2 fold change and a *q*-value cut-off of 0.05. The ten upregulated and downregulated proteins are indicated with their gene symbols: PDLIM3 (PDZ and LIM domain protein 3), COL4A5 (collagen alpha-5(IV) chain), CDH13 (cadherin-13), SCG2 (secretogranin-2), TGFB2 (transforming growth factor beta 2), PTX3 (pentraxin-related protein), DCN (decorin), PDGFD (platelet-derived growth factor D), C1R (complement C1r subcomponent), CFH (complement factor H), and CLU (clusterin). (**B**) STRING network formed by the proteins upregulated in MLC-OA sEVs, with the main cluster (elastic fiber formation and matrix metalloproteinases) shown in red. (**C**) STRING network formed by the proteins downregulated in MLC-OA sEVs, forming a closely interconnected cluster related to extracellular matrix (ECM) proteoglycans. Lines connecting the nodes (proteins) represent predicted protein–protein interactions. Different colors indicate the type of evidence supporting the interaction: green for gene neighborhood, blue for gene co-occurrence, purple for experimental evidence, black for co-expression, light blue for database evidence, and pink for text mining. Dashed lines represent interactions predicted by homology.

A total of 1229 proteins were detected in the MLC-derived sEVs, of which 62 (5%) were differentially expressed between the MLC-N sEVs and the MLC-OA sEVs, using a threshold of ±0.6 log_2_ fold change and a *q*-value cut-off of 0.05 (Figure 5A, Supplementary Table S2). To investigate the functional characteristics of MLC-derived sEVs, we conducted a functional enrichment analysis of all proteins detected in both groups using FunRich. In the cellular component category, “exosomes” emerged as the most statistically significant term (FDR-adjusted *p*-value < 10^−260^) and the most enriched term after “cytoplasm”. In the biological process category, the most significantly enriched terms were “cell growth”, “protein metabolism”, and “energy pathways”. Further enrichment analysis using Enrichr revealed additional significantly enriched biological terms, including immune system-related terms such as “extracellular matrix organization”, “collagen fibril organization”, and “regulation of cell migration”. In the cell ontology category, a strong positive association (odds ratio > 10, adjusted *p*-value < 10^−20^) was observed with MSCs from various tissues, including adipose tissue (CL:0002570) and muscle (CL:0000134), among others (CL:0008019) (Supplementary Table S3).

Of the 62 proteins differentially expressed, 27 were upregulated in the MLC-OA sEVs (Table 2), while 35 were downregulated (Table 3). STRING network analysis revealed that 20 of the 27 upregulated proteins formed a closely interconnected network (Figure 5B). The largest cluster identified by k-means clustering, comprising 10 of these 20 proteins, was associated with “elastic fiber formation and matrix metalloproteinases” (number of edges/expected number of edges: 22/1; protein–protein interaction (PPI) enrichment *p*-value: <10^−16^). Similarly, 15 of the 35 downregulated proteins in the MLC-N sEVs were also interconnected (Figure 5C), all belonging to a single cluster related to “extracellular matrix proteoglycans” (number of edges/expected number of edges: 25/2; PPI enrichment *p*-value: <10^−16^).

Importantly, 13 of the proteins upregulated in MLC-OA sEVs (48%) are known to be increased in OA. Among these, seven are associated with cartilage degradation: pentraxin-related protein (PTX3) [22], complement C1r subcomponent (C1R) [23], collagen alpha-3(VI) chain (COL6A3) [24], collagen alpha-1(VI) chain (COL6A1) [25], EGF-containing fibulin-like extracellular matrix protein 2 (EFEMP2) [26], matrix metalloproteinase-2 (MMP2) [27,28], and laminin subunit alpha-4 (LAMA) [25]. Conversely, four proteins exhibit chondroprotective or pro-chondrogenic effects: decorin (DCN) [29], biglycan (BGN) [29], clusterin (CLU) [30], chondroitin sulfate proteoglycan 4 (CSPG4) [31], and nidogen-2 (NID2) [32]. Microfibril-associated glycoprotein 4 (MFAP4) is also increased in OA patients, although its role in the pathology remains unclear [33] (Table 2). On the other hand, only one of the upregulated proteins (3.7%), sulfhydryl oxidase 1 (QSOX), has been reported to be decreased in OA, specifically in menisci [34].

**Table 2:** Proteins upregulated in MLC-OA sEVs compared to MLC-N sEVs.

UniProt	Protein	Ratio	*q*-Value	Potential Role in OA
P26022	PTX3	38.15	0.0450	Cartilage degradation [35]
P07585	DCN	25.37	0.0312	Cartilage protection [29,36]
Q9GZP0	PDGFD	14.28	0.0254	Unknown
P00736	C1R	11.59	0.0409	Cartilage degradation [37]
P08603	CFH	11.33	0.0409	Anti-inflammatory [38]
P12111	COL6A3	9.46	0.0466	Cartilage degradation [39]
P55083	MFAP4	8.36	0.0312	Vascular remodeling [33]
P12109	COL6A1	7.82	0.0254	Cartilage degradation [39]
Q96SM3	CPXM1	7.65	0.0466	Unknown
P10909	CLU	6.51	0.0001	Cartilage protection [30]
Q9UBX5	FBLN5	6.36	0.0439	Cartilage protection [40]
P21810	BGN	6.35	0.0157	Cartilage protection [29]
O00391	QSOX1	3.60	0.0312	Unknown
Q6UVK1	CSPG4	3.48	0.0312	Cartilage protection [41]
O43488	AKR7A2	3.42	0.0254	Unknown
Q14112	NID2	3.34	0.0411	Pro-chondrogenic [42]
P21399	ACO1	2.74	0.0362	Unknown
P25940	COL5A3	2.63	0.0362	Bone formation [43]
O95967	EFEMP2	2.53	0.0409	Cartilage degradation [44]
Q9UN67	PCDHB10	2.41	0.0493	Unknown
Q4LDE5	SVEP1	2.38	0.0466	Bone formation [45]
P01130	LDLR	2.33	0.0254	Unknown
P08253	MMP2	2.32	0.0439	Cartilage degradation [46]
Q16363	LAMA4	2.24	0.0409	Cartilage degradation [47,48]
P16035	TIMP2	2.12	0.0362	Cartilage protection [49,50]
O75718	CRTAP	1.84	0.0409	Bone formation [51]
Q9UHY1	NRBP1	1.69	0.0466	Unknown

In contrast, only three of the proteins downregulated in MLC-OA-sEVs (8.6%) have been reported to be increased in OA: secretogranin-2 (SCG2) [52], desmoplakin (DSP) [53], and vitronectin (VTN) [54]. Moreover, five of the downregulated proteins have been associated with beneficial effects in OA: transforming growth factor beta 2 (TGFB2) [55], high-mobility group protein HMGI-C (HMGA2) [56], integrin beta-1 (ITGB1) [57], cathepsin D (CTSD) [58], and phosphatidylethanolamine-binding protein 1 (PEBP1) [59]. Among these, ITGB1 has been shown to be decreased in OA joint tissues [57], while CTSD and PEBP1 are specifically reduced in OA cartilage [60,61]. Notably, unlike the upregulated proteins, most of the downregulated proteins lack a clear role in cartilage biology or OA pathology (Table 3). Statistical analysis using a contingency table revealed a significant association between protein regulation status (upregulated vs. downregulated) and their known association with OA (Fisher’s exact test *p*-value = 0.0117; chi-square test *p*-value = 0.0094).

**Table 3:** Proteins downregulated in MLC-OA sEVs compared to MLC-N sEVs.

UniProt	Protein	Ratio	*q*-Value	Potential Role in OA
Q53GG5	PDLIM3	0.0768	0.0439	Unknown
P29400	COL4A5	0.1039	0.0312	Unknown
P55290	CDH13	0.1314	0.0342	Inhibition of osteoclast differentiation [62]
P13521	SCG2	0.1400	0.0439	Anoikis [52]
P61812	TGFB2	0.1626	0.0415	Pro-chondrogenic [55]
Q9Y281	CFL2	0.2079	0.0466	Unknown
Q8N6G6	ADAMTSL1	0.2098	0.0312	Chondro-proliferative [63]
P52926	HMGA2	0.2297	0.0254	Cartilage protection [56]
P30837	ALDH1B1	0.2525	0.0015	Unknown
P12429	ANXA3	0.2628	0.0470	Unknown
P05556	ITGB1	0.2993	0.0254	Cartilage protection [57]
P15586	GNS	0.3035	0.0362	Unknown
O95721	SNAP29	0.3231	0.0486	Unknown
P21980	TGM2	0.3324	0.0312	Unknown
Q15942	ZYX	0.3400	0.0466	Unknown
O43155	FLRT2	0.3588	0.0312	Pro-chondrogenic [64]
P07339	CTSD	0.3784	0.0312	Pro-autophagic [58]
P78324	SIRPA	0.4186	0.0312	Unknown
P35527	KRT9	0.4252	0.0098	Unknown
Q99439	CNN2	0.4314	0.0065	Unknown
O75083	WDR1	0.4349	0.0450	Unknown
P15924	DSP	0.4353	0.0466	Unknown
P07602	PSAP	0.4385	0.0342	Unknown
P29692	EEF1D	0.4462	0.0409	Unknown
P30086	PEBP1	0.4510	0.0312	Anti-ferroptotic [59]
P04264	KRT1	0.4681	0.0312	Unknown
P27816	MAP4	0.4691	0.0466	Unknown
P37802	TAGLN2	0.4751	0.0439	Unknown
P40123	CAP2	0.4811	0.0254	Unknown
P04004	VTN	0.5033	0.0466	Inhibition of osteoclast activity [65,66]
P10155	RO60	0.6230	0.0466	Unknown

## 3. Discussion

OA is a prevalent, age-related joint disorder characterized by the progressive degeneration of articular cartilage, resulting in pain, stiffness, and impaired mobility. Despite significant research efforts, effective therapies for regenerating cartilage remain elusive [5]. In this context, MSC-derived sEVs have garnered considerable interest due to their regenerative and immunomodulatory properties [6,7,8]. Induced pluripotent stem cell (iPSC)-derived MLCs offer a promising alternative to primary MSCs, providing better scalability and enhanced paracrine activity [11,14,15]. Notably, sEVs derived from MLCs have demonstrated chondroprotective effects similar to those of MSC-derived sEVs [16,17,18,19]. Given that sEVs derived from autologous MSCs have been demonstrated to have chondroprotective effects [10,11], we decided to investigate the feasibility of using MLCs as a source of autologous sEVs for OA therapy.

MLCs derived from both healthy and OA iPSC lines exhibited robust mesenchymal phenotypes, as demonstrated by mesenchymal marker expression, osteogenic and chondrogenic differentiation potential, and the enrichment of MSC-related terms in the protein content of their sEVs. Both MLC-N and MLC-OA showed a high expression of all the surface markers typically included in the MSC panel, except for CD105. Since CD105 expression is known to decrease with passaging in primary MSCs [67], this is possibly due to the processes of MLC differentiation and subculturing. Intriguingly, both MLC types were capable of differentiating into osteoblasts and chondrocytes but failed to differentiate into adipocytes. Osteogenesis and adipogenesis are opposing and mutually controlled processes [68,69], and thus cells with a stronger osteochondrogenic commitment may require longer exposure times or additional *stimuli* to produce mature adipocytes. Interestingly, MSCs have been reported to become more adipogenic during in vitro aging [70], suggesting that a stronger osteochondrogenic commitment might reflect the proliferative, “rejuvenated” state of MLCs.

Both types of MLCs secreted sEVs in comparable yields, with MLC-OA sEVs being slightly larger but remaining within the typical size range of sEVs (<200 nm). Proteomic analysis revealed that the cargo of MLC-derived sEVs was enriched in processes related to extracellular matrix organization and immune system regulation, suggesting a potential role in modulating cartilage homeostasis and inflammation. The analysis of differentially expressed proteins revealed a high abundance of OA-associated proteins among those upregulated in the MLC-OA sEVs. The most upregulated protein in the MLC-OA sEVs was PTX3, which plays a critical role in regulating inflammation and tissue remodeling in OA. Elevated levels of PTX3 in the joint microenvironment correlate with increased M1 macrophage polarization, promoting a feedback loop between macrophages and chondrocytes that exacerbates cartilage damage. In addition, the neutralization of PTX3 has been shown to alleviate synovitis and cartilage degeneration [35]. Furthermore, elevated serum PTX3 levels have been proposed as a biomarker for OA, demonstrating significant sensitivity and specificity in distinguishing OA patients from healthy controls [22].

Other upregulated proteins included MFAP4, which has been associated with OA-related vascular changes [33]; EFEMP2, which reduces extracellular matrix production and suppresses chondrocyte differentiation [44]; and LAMA4, which promotes MMP-13 expression and chondrocyte cluster formation [47,48]. Interestingly, several other highly upregulated proteins have been associated with OA pathology but are reported to have chondroprotective effects. For instance, DCN and BGN are upregulated during late stages of OA, potentially as part of a compensatory response to counteract the proteoglycan loss characteristic of advanced disease stages [29]. Similarly, NID2, which shows higher expression in OA chondrocytes [32], enhances the expression of chondrogenic factors [42]. Likewise, the upregulation of metalloproteinase inhibitor 2 (TIMP2) may reflect a compensatory mechanism aimed at counterbalancing the increased expression of MMP2.

In contrast, most of the proteins downregulated in MLC-OA sEVs did not have clear roles in OA or cartilage homeostasis. The most downregulated protein, PDZ and LIM domain protein 3 (PDLIM3), is highly expressed in skeletal muscle and associated with Hedgehog signaling [71]. Differential levels of PDLIM3 may reflect variations in cytoskeletal organization or the differentiation status of the parent cells. Another significantly downregulated protein, cadherin-13 (CDH13), inhibits osteoclast differentiation and prevents age-related bone loss [62]. Similarly, vitronectin (VTN), another downregulated protein, inhibits osteoclast activity and, specifically, osteoclast-mediated collagen degradation [65,66]. Taken together with the upregulation of several proteins related to bone formation, these findings may indicate a connection to the increased bone turnover commonly associated with OA [72].

Other downregulated proteins have been associated with chondrogenic effects. TGFB2 inhibits the expression of matrix metalloproteinases, pro-inflammatory cytokines, and the hypertrophic marker type 10 collagen (COL10A1) in human OA articular cartilage explants [73], and its silencing suppresses the expression of early chondrogenic markers in chondrogenic cells [55]. Likewise, HMGA2, a chromatin remodeling factor, binds to the SOX9 promoter, increasing its expression and promoting chondrogenesis [56]. Interestingly, HMGA2 also regulates the balance between the self-renewal and differentiation of stem cells, contributing to the “rejuvenated” state of MLCs compared to primary MSCs [74]. Similarly, ITGB1 overexpression reduces pro-inflammatory cytokine levels and inhibits chondrocyte apoptosis [57]. In addition, MSC-derived sEVs have been shown to promote chondrocyte proliferation and differentiation by transferring ITGB1, which activates the TGF-β/Smad2/3 axis [75].

The expression of protein-glutamine gamma-glutamyltransferase 2 (TGM2) has also been linked to chondrogenic differentiation, and its expression levels in unstimulated MSCs correlate with proteoglycan levels after chondrogenic induction [76]. Furthermore, TGM2 is downregulated in senescent MSCs and plays a role in autophagy [77]. Similarly, the leucine-rich repeat transmembrane protein FLRT2 promotes MSC chondrogenesis [64] and is inhibited by inflammatory *stimuli* [78], while CTSD is upregulated during the transformation of prochondroblasts into chondroblasts [79] and is also highly expressed in repair chondrocytes [80]. Zyxin (ZYX) is involved in the synthesis of collagen and the remodeling of chondrocyte cytoskeleton–extracellular matrix adhesion, and its knockdown inhibits the expression of type II and type X collagen [81]. In contrast, TAGLN2 downregulation is associated with the chondrogenic differentiation of MSCs [82].

In conclusion, these findings suggest that, despite cellular reprogramming, iPSC-derived MLCs and their sEVs retain the OA-specific protein signatures of the donor. The presence of proteins associated with cartilage degradation in autologous MLC-derived sEVs may negatively impact their regenerative and immunomodulatory potential, thus limiting their therapeutic efficacy. This contrasts with the findings of Sanjurjo et al. (2021), who demonstrated that sEVs derived from MSCs of OA patients could enhance chondrocyte viability and proliferation similarly to sEVs derived from MSCs of healthy donors [10]. A limitation of this study is the small sample size, and further analysis of samples from additional patients will be necessary to validate and confirm the proteomic findings. In addition, further research is needed to evaluate the impact of the enrichment of OA-specific proteins on the functionality and therapeutic potential of autologous MSC- or MLC-derived sEVs, including their effects on chondrocyte viability, metabolism, and inflammation. This study underscores the importance of carefully evaluating disease-specific protein profiles in sEVs for regenerative applications.

## 4. Materials and Methods

### 4.1. Culture and Differentiation of iPSCs

This study was reviewed and approved by the ethics committee of research from A Coruña-Ferrol, Spain (2020/477). Two iPSC lines previously generated, one from an OA patient [20] and one from a donor with no rheumatic diseases [21], were cultured in mTeSR medium (StemCell Technologies, Vancouver, BC, Canada) on Matrigel Matrix-coated culture plates (Corning Life Sciences, Durham, NC, USA) and differentiated into MLCs via embryoid body (EB) formation. Briefly, iPSC colonies were mechanically detached and cultured as EBs in non-adherent culture dishes in mTeSR medium for three days, with the medium being renewed every day. EBs were thereafter seeded on adherent culture plates pre-coated with EmbryoMax 0.1% Gelatin Solution (Millipore, Sigma-Aldrich Química S.A., Madrid, Spain) and cultured in EB medium, consisting of DMEM Knockout with 20% fetal bovine serum (FBS), 1% non-essential aminoacids, 1% Glutamax 100X, 1% penicillin/streptomycin (P/S), and 0.1 mM β-mercaptoethanol (all from Gibco, Thermo Fisher Scientific, Madrid, Spain). After enough cells had grown out of the EBs, subculturing was performed with 0.25% trypsin-EDTA (Gibco). The EB medium was renewed twice a week.

### 4.2. Flow Cytometry

The expression of surface markers of MSCs (CD29, CD44, CD73, CD90 and CD105) and hematopoietic stem cells (CD34 and CD45) was analyzed by flow cytometry, as previously described [13], employing the fluorescent-labeled antibodies and isotype controls listed in Table 4. Data acquisition was carried out using a CytoFLEX flow cytometer (Beckman Coulter, Barcelona, Spain), and the data obtained were analyzed using CytExpert software (https://www.beckman.com/flow-cytometry/research-flow-cytometers/cytoflex/software) (Beckman Coulter). The results are shown as the mean percentage of positive cells in three different passages.

### 4.3. Tri-Lineage Differentiation of MLCs

MLCs were differentiated into osteoblasts, adipocytes, and chondrocytes in order to assess their multi-differentiation potential. For osteogenic and adipogenic cell differentiation experiments, 10^4^ cells were plated on 8-well chamber slides and 10^5^ cells were plated on 6-well plates to perform histological and gene expression analyses, respectively. Cells were grown for 21 days using the *StemPro Ostogenesis Differentiation Kit* (Gibco) for osteoblastogenic differentiation and the *hMSC Adipogenic Differentiation BulletKit*™ *Medium* (Lonza, Madrid, Spain) for adipogenic differentiation, and the medium was renewed every 3 days. For chondrogenic cell differentiation experiments, cell aggregates of 2.5 × 10^5^ cells were formed by centrifugation and incubated in *Mesenchymal Stem Cell Chondrogenic Differentiation Medium* (PromoCell, Heidelberg, Germany) supplemented with 10 ng/mL of human transforming growth factor β-3 (TGF-β3) (ProSpec-Tany TechnoGene, Rejovot, Israel) for 21 days, and the medium was renewed twice a week.

### 4.4. Histological Analysis

After osteogenic differentiation, cells were fixed with 4% paraformaldehyde and stained with Alizarin Red, and slides were mounted with DPX mounting medium (Surgipath, Leica Microsistemas S.L., Barcelona, Spain). Adipogenically differentiated cells were fixed with 4% paraformaldehyde and stained with Oil Red O, and slides were mounted with Glycergel aqueous mounting medium. Chondrogenically induced cell aggregates were fixed with 3.7% formaldehyde (Panreac Química S.L.U., Barcelona, Spain), embedded in paraffin (Millipore), cut in a microtome, and stained with Masson’s Trichrome and Safranin O. Slides were observed by employing an Olympus BX61 microscope coupled to an Olympus DP70 digital camera. Micrographs were obtained using the cellSens Dimension software (Olympus Life Science, Waltham, MA, USA).

### 4.5. Gene Expression Analysis

RNA was isolated by employing TRIzol Reagent (Thermo Fisher Scientific) and chloroform, precipitated with isopropanol, and washed with ethanol (all from Sigma-Aldrich Química S.A.). Reverse transcription of 500 ng of RNA was carried out using the SuperScript VILO cDNA Synthesis kit, following the manufacturer’s instructions (Thermo Fisher Scientific), in an Applied Biosystems Veriti 96-Well Thermal Cycler (Thermo Fisher Scientific). Quantitative real-time polymerase chain reaction (qPCR) was performed in a LightCycler 480 Instrument (Roche, Basel, Switzerland), employing LightCycler 480 SYBR Green I Master (Roche) in addition to the gene-specific primers shown in Table 5.

Data analysis was performed using LightCycler 480 Relative Quantification software (Roche), and relative gene expression levels (RELs) were calculated by employing qbase+ software (Biogazelle, Zwijnaarde, Belgium). RELs were normalized to undifferentiated cells and are shown as the mean ± standard error. YWHAZ was employed as the reference gene.

### 4.6. Isolation of Small Extracellular Vesicles

MLCs were seeded in 150 mm diameter adherent culture dishes. When they reached 90% confluence, MLCs were washed twice with PBS and cultured in serum-free EB medium (15 mL/dish). After 72 h, the culture supernatant was collected, filtered through a 0.22 pore filter, and incubated with ½ volume Total Exosome Isolation Reagent (Invitrogen, Thermo Fisher Scientific) overnight at 4 °C, following the manufacturer’s instructions. This mixture was thereafter centrifuged at 3.900× *g* for 180 min at 4 °C to obtain sEV pellets.

### 4.7. Western Blotting

Proteins were isolated from pelleted sEVs using Pierce™ RIPA buffer with Halt™ Protease and Phosphatase Inhibitor Cocktail (Thermo Fisher Scientific) and loaded onto a 10% sodium dodecyl sulfate polyacrylamide gel. After electrophoresis (SDS-PAGE), proteins were transferred to a polyvinylidene difluoride (PVDF) membrane (Millipore) and incubated at 4 °C overnight with mouse primary antibodies anti-human CD9 and CD63 (1:250), both from BD Pharmigen (Franklin Lakes, NJ, USA). Incubation with the secondary antibody ECL HRP-Linked Anti-mouse IgG (1:1000; NA931V, GE HealthCare, Chicago, IL, USA) was performed for 1 h at room temperature. Target proteins were visualized on an Amersham Imager 600 (GE HealthCare) employing Clarity™ Western ECL Substrate (BioRad, Hercules, CA, USA).

### 4.8. Transmission Electron Microscopy

Transmission electron microscopy (TEM) was performed by the Microscopy Unit of the Research Support Services from Universidade da Coruña (SAI-UDC). Briefly, one drop of PBS-suspended sEVs was absorbed on carbon-coated grids for 5 min. Then, excess liquid was removed with filter paper, and negative staining was performed with 1% uranyl acetate for 30 s. Samples were observed on a JEOL JEM-1010 (JEOL Ltd., Akishima, Japan) at 100 kV.

### 4.9. Nanoparticle Tracking Analysis

Nanoparticle tracking analysis (NTA) was performed by the Biomaterial Processing and Nanostructuring Unit (SOFT/U6 Nanbiosis) of the Institut de Ciència de Materials de Barcelona (ICMAB-CSIC). Briefly, sEV pellets were diluted in PBS (50 µL for each 30 mL of conditioned media), diluted to 1:20, and analyzed on a Nanosight NS300 (Malvern Panalytical, Malvern, UK).

### 4.10. Proteomic Analysis

Proteomic analysis was performed by the Proteomics Unit of the Interdisciplinary Center for Chemistry and Biology (CICA) at Universidade da Coruña (UDC). For this analysis, sEVs were isolated from MLC-N and MLC-OA at three different passages. Nano-liquid chromatography–mass spectrometry was performed using a nanoElute nano-flow liquid chromatography system coupled to a high-resolution trapped ion-mobility quadrupole time-of-flight (TIMS-QTOF) mass spectrometer (timsTOF Pro, Bruker Daltonics) with a CaptiveSpray ion source (Bruker Daltonics, Billerica, MA, USA). Peptides were analyzed through data-independent acquisition (DIA) with Parallel Accumulation–Serial Fragmentation (PASEF) enabled. DIA-PASEF raw files were processed in Spectronaut software v18.3 (Biognosys AG, Schlieren, Switzerland) in library free mode. Protein identification was performed by comparing against the huma UniProtKB/Swis-Prot database.

Only changes with a log_2_ fold change threshold of ±0.6 and a *q*-value cut-off of ≤0.05 were considered statistically significant. Enrichment and protein–protein interaction analyses were performed using FunRich version 3.1.3 [83], Enrichr (https://maayanlab.cloud/Enrichr/) [84], and STRING version 12.0 [85]. A volcano plot was created with VolcaNoseR [86]. For each differentially expressed protein, Biotools’ Pubmed Multiple Keyword Search tool was used with the keywords “osteoarthritis”, “cartilage”, and “chondrocyte”.

## Figures and Tables

**Table 1:** Mean percentage expression of CD29, CD44, CD73, CD90, CD105, CD34, and CD45 in MLC-N and MLC-OA across three passages, as measured by flow cytometry.

Cells	CD29	CD44	CD73	CD90	CD105	CD34	CD45
MLC-N	94.61%	95.52%	80.56%	93.30%	38.20%	1.63%	2.18%
MLC-OA	93.06%	94.49%	82.85%	84.50%	29.29%	0.72%	2.08%

**Table 4:** Antibodies conjugated with fluorescein isothiocyanate (FITC), phycoerythrin (PE), or PE/Cy5 used for flow cytometry.

Antibody	Dilution	Specificity	Clone	Source
FITC Mouse IgG1 Isotype Control	1:50	-	ICIG1	Immunostep
PE Mouse IgG1 Isotype Control	1:50	-	B11/6	Immunostep
PECy5 Mouse IgG1 Isotype Control	2:25	-	1F8	Abcam
PE Mouse Anti-Human CD29	3:50	Human integrin β1 (ITGB1)	VJ1/14	Immunostep
PE Mouse Anti-Human CD34	2:25	Hematopoietic progenitor cell antigen 1 (HPCA1)	581	BD Pharmingen
FITC Mouse Anti-Human CD44	1:50	Homing cellular adhesion molecule (HCAM)	IM7	BD Pharmingen
FITC Mouse Anti-Human CD45	3:50	Leukocyte common antigen (LCA)	D3/9	Immunostep
PE Mouse Anti-Human CD73	3:50	Ecto-5′-nucleotidase (NT5E)	AD2	Immunostep
PECy5 Mouse Anti-Human CD90	1:50	Thymocyte differentiation antigen 1 (Thy-1)	5E10	Immunostep
FITC Mouse Anti-Human CD105	1:50	Human Endoglin (ENG)	SN6	AbD Serotec

**Table 5:** Primers employed for quantitative real-time PCR (qPCR) analysis.

Gene	Forward Primer 5′→3′	Reverse Primer 5′→3′
Tyrosine 3-monooxygenase/tryptophan 5-monooxygenase activation protein zeta (YWHAZ)	GATCCCCAATGCTTCACAAG	TGCTTGTTGTGACTGATCGAC
Homosapien Runt-related transcription factor 2 (RUNX2)	TTACTTACACCCCGCCAGTC	TATGGAGTGCTGCTGGTCTG
Alkaline phosphatase, biomineralization associated (ALPL)	GACGGACCCGTCACTCTC	GTGCCCGTGGTCAATTCT

## Data Availability

The data used to support the findings of this study are contained within the article. Raw data are available from the corresponding author upon request.

## Supplementary Materials

The supporting information can be downloaded at https://www.mdpi.com/article/10.3390/ijms26030870/s1.
